# Care Coordination and Patient Satisfaction With Ambulatory Cancer Care During the COVID-19 Pandemic in Manitoba, Canada: Report of An Online Survey Study of Patient-Reported Experience Measures With Interpretation Guided by Fit Theory

**DOI:** 10.2196/58999

**Published:** 2025-08-25

**Authors:** Maclean Thiessen, Andrea Soriano, Jason Park, Kathleen Decker

**Affiliations:** 1 Department of Medical Oncology and Hematology, CancerCare Manitoba, L1 101-13, 409 Tache Ave, Winnipeg, MB, R2H 2A6, Canada, 1 204 237 2472, 1 204 237 6048; 2 Department of Internal Medicine, Max Rady College of Medicine, University of Manitoba, Winnipeg, MB, Canada; 3 Department of Surgery, University of British Columbia, Vancouver, BC, Canada; 4 Department of Community Health Sciences, Max Rady College of Medicine, University of Manitoba, Winnipeg, MB, Canada; 5 Paul Albrechtsen Research Institute, CancerCare Manitoba, Winnipeg, MB, Canada

**Keywords:** data collection, surveys and questionnaires, quality of health care, patient satisfaction, covid-19, grounded theory, cancer, ambulatory care, online survey, patient-reported measures, patient experience, mixed methods

## Abstract

**Background:**

In Manitoba, Canada, the impact of the COVID-19 pandemic on cancer care delivery included, but was not limited to, disruption of many routine health care services, and the rapid introduction of both social distancing and virtual care. Little was known about how COVID–19-related changes to cancer care delivery would impact patient satisfaction with care and care coordination.

**Objective:**

This report aims to present and interpret findings of an online survey of people with oncology-related conditions in Manitoba, Canada, during the COVID-19 pandemic, exploring patient satisfaction and care coordination.

**Methods:**

A link to an online survey was made available to patients receiving cancer treatment in Manitoba, Canada, between July 31, 2020, and February 28, 2022. The survey included validated patient-reported experience measures (PREMs) for patient satisfaction and care coordination. Analysis included the generation of descriptive statistics and logistic regression, including univariate and multivariate analysis using stepwise model building. The survey results were interpreted using fit theory as a theoretical lens.

**Results:**

A total of 203 responses were collected, of which 154 had completed responses for all PREM measures and were included in the analysis. Response rate is estimated at 3.3%‐2.0%. The average age was 65 (SD 11.7) years. Most respondents were male (n=79, 52.7%). Most respondents were being treated with curative intent (n=81, 53.6%). The most common type of cancer was breast (n=41, 26.6%). Univariate analysis demonstrated that ages 60‐69 years were associated with above average patient satisfaction (OR 2.205, 95% CI 1.045‐4.624; *P*=.04). Age <60 years (OR 0.437, 95% CI 0.204‐0.934; *P*=.03) and European Cooperative Group functional status (ECOG) ≥2 (OR 0.327, 95% CI 0.137‐0.782; *P*=.01) were associated with below average patient satisfaction. Age <60 years, ECOG ≥2, and hematological cancer were selected through stepwise multivariate model building, resulting in an explanatory model (*R*^2^=0.129) of patient satisfaction. ECOG ≥2 was associated with below-average care coordination (OR 0.357, 95% CI 0.145‐0.880; *P*=.03), and was the only identified predictor of care coordination, with no explanatory multivariate model generated. Fit theory supports that the level of patient satisfaction and care coordination in each population subset inversely reflects a relative level of unmet supportive care need.

**Conclusions:**

Survey respondents with poor functional status, those outside the 60‐69 years age range, and those with nonhematological malignancies likely experience increased unmet supportive care needs compared with their counterparts. Further research is needed to determine whether these findings reflect transient phenomena related to the COVID-19 pandemic, selection biases associated with the survey method used, or underlying health care delivery inequities.

## Introduction

### Background

In the province of Manitoba, Canada, the dynamic state of cancer care delivery early in the COVID-19 pandemic was considered an opportunity to better understand the cancer experience [1-3]. The many changes to service delivery resulting from the pandemic [4], including the implementation of virtual care, limitations on the number of care partners able to accompany a patient to ambulatory visits, and changes to the availability of nonemergent diagnostic services, all had the potential to impact both the experience of receiving care and living with cancer.

### Need for Cancer Experience Data and Theory

Ideally, a robust system for collecting real-world cancer experience data (eg, patient-reported experience measures or PREMs) capable of measuring changes in real-time would have been in place prepandemic [5]. This data could have provided powerful insights for real-world problem solving and improving the cancer journey. However, as is the case in many cancer centers [6], strategies for collecting the patient-reported measures specifically designed to understand the cancer experience are just beginning to evolve.

Similarly, theory and theory-based tools are lacking for interpreting experience data collected from people diagnosed with cancer and their supporters. One patient experience framework published in 2020 provides a general, high-level, approach to understanding the patient experience [7]. The framework presents several useful concepts, including important distinctions between person, patient, and health care services user, all of which have considerable face validity. However, the framework is not cancer-specific and is limited in how it addresses the more granular nuances of the experience of living with cancer and receiving cancer care. Middle-range theories are better suited to assist with the interpretation of data in the cancer context and directly inform cancer-specific clinical practice and research [8,9]. While a systematically conducted literature review would be helpful, it appears that such theories of the cancer patient experience are absent from the literature.

### Understanding the Experience of Living With Cancer Using an Online Survey

To better understand the cancer experience during the pandemic in Manitoba, Canada, an electronic survey of people diagnosed with cancer was conducted. Recruitment for the electronic survey was integrated into clinical care to collect ongoing real-world experience data. The survey included question items on satisfaction with ambulatory cancer care and care coordination using validated PREM questionnaires [10,11]. Satisfaction with care was chosen because it was hypothesized that, due to the rapid adoption of virtual care in the province, people diagnosed with cancers may be less satisfied with their interactions with the health care team because of less face-to-face contact. Coordination of care was chosen because it was hypothesized that the changes in health care delivery that occurred in the province at the start of the pandemic, such as limiting when support partners could participate in ambulatory care visits - eliminating the benefit of the “extra set of ears” [12] that support partners provide - would impact patients’ perceptions of how well care was coordinated. This survey was conducted alongside the development of a middle-range classic grounded theory of the cancer experience titled fit theory [1].

Initially, the intention was to repeat the survey once COVID-19–related health care delivery changes were rolled back and compare results. However, years after the start of the pandemic, many of the changes, such as the implementation of virtual care, remain in place, making the value of repeating the survey outside of routine quality assurance work less clear. However, reporting on the survey data that was collected is likely to be of scientific value given that (1) the data was collected during the COVID-19 pandemic and (2) the use of validated PREMs facilitates comparison with previously conducted work at other centers. In addition, the survey provides a unique opportunity to apply fit theory [1] as a tool to gain a deeper understanding of the collected data.

### Objectives

This report presents the findings of the survey, namely how patient satisfaction and care coordination differ among the cancer population surveyed in Manitoba, Canada, during the COVID-19 pandemic, as well as an interpretation of the survey findings guided by fit theory [1].

## Methods

### Study Context

The research study was conducted in the province of Manitoba, Canada. Manitoba is a province with a geographic area of 650,000 km^2^ [13] and a population of 1.3 million people [14]. Cancer care services within the province are delivered by CancerCare Manitoba. Specialty services are largely centralized in Winnipeg, Manitoba, the province’s capital and largest city. However, a number of smaller community cancer sites offering systemic therapy exist throughout the province in both rural and remote areas [15]. Radiotherapy treatment centers are located only in the cities of Winnipeg and Brandon, Manitoba.

The survey was conducted, in part, to facilitate recruitment and participant selection for the associated classic grounded theory study, which aimed to develop an explanatory and predictive theory of the cancer experience. Classic grounded theory relies on purposive and theoretical sampling [16]. By linking the survey study to the classic grounded theory study, the researchers were able to purposively select individuals for semistructured interviews in the classic grounded theory study based on their survey responses (ie, demographic and PREM responses), ensuring that contrasting cases were selected. A complete description of fit theory, including its development as well as a brief positionality statement of the lead author, is reported elsewhere [1].

### Survey Instrument

The survey was developed using an online survey solution (Enterprise account, SurveyMonkey, One Curiosity Way) meeting the standards of the Health Information Portability and Accountability Act (HIPAA). Survey development and reporting in this manuscript were guided by the Checklist for Reporting Results of Internet E-Surveys (CHERRIES) [17]. The survey was initially developed by the lead author with additional revisions incorporated after review by co-authors KD, JP, and AS. It was not piloted or pretested beyond the review by the co-authors. See Checklist 1 for the completed CHERRIES checklist.

The first page of the survey outlined the survey purpose, investigators, voluntary nature of the study, associated risks and benefits with participating, and expected time to complete the survey. Participants were then asked to confirm that they were 18 years of age and older and provide their medical record number. Next, respondents were asked to respond to 2 validated PREMs focusing on satisfaction with ambulatory oncology care [11] and experience with care coordination [10], followed by researcher-generated items related to the use of virtual care. Respondents were then asked to enter their postal code, date of birth, gender, marital status, treatment intent, cancer type, time since diagnosis, and self-reported functional status using a modified version of the European Cooperative Oncology Group (ECOG) functional scale [18]. Finally, respondents were provided the opportunity to opt in to being contacted about additional research opportunities. Each survey page contained no more than 5 question items, with most pages containing between 3 and 5 items. The survey was a total of 16 pages. The survey was divided into 6 sections and contained 60 questions.

One response was allowed per digital device. Participants were able to review and change their responses before submission. Paper versions of the survey were also prepared to ensure accessibility to the study for those not wishing to participate electronically. These were made available to participants upon request from the clinical teams at the treatment locations. Upon their completion, these paper versions were faxed to the researchers and the responses were manually entered into the survey dataset by MT. See Multimedia Appendix 1 for the survey tool.

The specific PREMs used in this survey included the Patient Satisfaction with Cancer Care (PSCC) and Cancer Care Coordination Questionnaire for Patients (CCCQP). The CCCQP is a validated cancer-specific tool for assessing the experience of care coordination. It consists of 2 subscales, communication (CCCQP_comm_) and navigation (CCCQP_nav_), and has demonstrated construct validity in testing that involved a sample (n=686) of people diagnosed with varying cancer types, primarily in Australia [10]. The PSCC is a validated cancer-specific tool for assessing satisfaction with oncology care in the ambulatory setting [11]. It consists of 1 domain and has demonstrated construct validity in testing that involved a sample (n=843) of people diagnosed with cancer of varying types, primarily in the United States [11]. Permission to use the validated PREM tools was obtained from the lead author of the manuscripts reporting on the development of each PREM [10,11].

### Data Collection

Based on an estimated 45% response rate [19], with ~456 patients treated monthly throughout the province, it was estimated that 209 surveys would be needed for a 95% CI with a margin of error of 5% after 1 month. Given the uncertainty regarding the response rates for the novel method used for recruitment, and the fact that the survey was being conducted at least in part to support recruitment for the co-occurring grounded theory study, the survey remained open until the classic grounded theory study completed recruitment.

Recruitment used a convenience sampling approach. An invitation to participate in an online survey was distributed to patients receiving intravenous and radiotherapy through CancerCare Manitoba using three methods, including: (1) posters placed in the waiting areas of the intravenous and radiotherapy treatment sites in the province, (2) an invitation message printed on the personalized appointment schedule that all patients receiving intravenous or radiotherapy receive, and (3) through invitation cards that were distributed to patients by nursing staff in treatment areas. Each recruitment method included a brief explanation of the survey and a link to complete the survey online. The link that was provided was unique to each of the 24 treatment sites where the recruitment material was being distributed. This allowed survey responses to be linked back to each of the treatment locations in Manitoba. The survey was not distributed through emails or publicly available on the internet. A password was not required to access the survey. No incentives were offered for participation.

Initially, all recruitment methods were used. However, 3 months after the survey opened, the invitation cards were asked to be returned to the researchers as it was not clear whether they were being handed out consistently across all centers. Survey responses were collected from July 31, 2020, to February 28, 2022, with the survey closing when recruitment for the associated grounded theory study was completed. Examples of the recruitment materials are included in Multimedia Appendix 2.

### Primary Outcomes

Outcomes for the study were to identify which population subsets, defined by age, gender, marital status, treatment location, performance status, cancer type, and treatment intent, experienced below and above average patient satisfaction and care coordination. The PREMs used in this survey do not have established cut-offs for adequate satisfaction or care coordination and evaluation of scores has taken a variety of approaches throughout the literature [20-23]. The use of below and above average PREMs scores as outcomes was informed by fit theory [1], as this theory supports that health care services will be a better or worse fit depending on the assets available and the characteristics of the patient.

### Data Analysis

Data analysis was conducted using IBM SPSS Statistics (Version 27, IBM Corp). Descriptive statistics were generated to summarize respondents’ characteristics, including age, cancer type, treatment intent, and functional status. Binary logistic regression was used to determine the correlation between above and below average satisfaction with care for both PREMs (including CCCQP_comm_ and CCCQP_nav_ subscales) used in the survey. Nondichotomous categorical outcomes were recoded into dichotomous outcomes using dummy variables for the purposes of analysis. After data recoding, testing for outliers, multicollinearity, and linearity was performed, and assumptions of binary logistic regression were determined to be satisfied. Univariate analysis and multivariate analysis were then performed. Given the exploratory nature of this analysis and the fact that the dependent variables were selected primarily to assist in selecting participants for the associated classic grounded theory study, multivariate analysis used stepwise model building. Liberal criteria for variable selection were chosen (ie, *P_in_*<.10, *P_out_*>.10) given the relatively small sample size compared with the number of variables being explored as well as the exploratory nature of this survey [24]. Only responses where all the items for the PREMs had been completed were included. Incomplete responses to one of the demographic or cancer characteristic questions did not exclude the respondents’ survey responses from being included in the analysis.

The response rate for this study was estimated by dividing the total number of responses (numerator) by an estimate of the total number of unique patients receiving systemic and radiation treatment during the study period (denominator). The estimate of the total number of unique patients receiving systemic treatment (n=7662) and radiation treatment (n=5352) was based on data publicly available from CancerCare Manitoba for the 24-month period from 2014‐2016 [15], adjusted to reflect a 19-month period, which is the amount of time the survey was open. As it is not clear from available data the exact number of patients receiving both systemic and radiation treatment, the response rate is reported as a range. The denominator for the maximum response rate excludes the patients undergoing radiation therapy, assuming that all patients undergoing radiation therapy also received systemic therapy and should not be counted twice (ie, denominator_max_=6066=(7662×19)/24). The denominator for the minimum response rate is the straight sum of both types of patients undergoing radiation and systemic therapy treatment, assuming that not all patients undergoing radiation therapy are receiving systemic therapy, and therefore all participants would only receive a link for the survey in one setting (denominator_min_=10292=((7662+5338)×19)/24).

### Interpretation of Quantitative Survey Data Using Fit Theory

Fit theory [1] was used as a tool to interpret the survey results. First, the individual concepts described by the theory [1] were reviewed by MT to determine how the statistically significant survey variables could be linked back to the theory. If a survey variable was referenced directly in the description of a concept in the fit theory manuscript [1] or could plausibly be directly linked to a concept defined by fit theory but not directly referenced, the concept was used to inform an explanation of the relationship between the survey variable and the PREM outcome. Where it was not possible to directly link a fit theory concept [1] to a statistically significant survey variable, an explanatory theoretical relationship was not developed.

### Ethical Considerations

Approval for this study, including the study procedures that were followed, was obtained from both the University of Manitoba Ethics Board (HREB: HS23979 [H2020:264]), and the CancerCare Manitoba Research Resource Impact Committee (RRIC: 2020‐14) before initiation of all study procedures. The initial page of the survey outlined the risks and benefits of participating in the survey, the confidential nature of the survey, as well as the potential risk of a privacy breach. Informed consent was implied if participants provided survey responses. While the survey did collect identifying data (ie, health record number and contact information), these data were removed before analysis. Raw data, including that which could be used to identify specific respondents, were not shared outside of the research team. Respondents did not receive compensation for study participation.

## Results

### Overview

Table 1 provides a summary of the respondent characteristics. PREMs outcomes, including average scores and number of respondents with above and below average scores, are summarized in Table 2. The univariate analysis of collected PREMS is presented in Table 3. Results of stepwise multivariate model building for satisfaction with care (PSCC) are presented in Table 4. No multivariate model resulted from stepwise model building for the care-coordination measure (CCCQP) or its subscales (CCCQP_comm_, CCCQP_nav_).

**Table 1.** Participant demographics (n=154).

Characteristic		Participants
Age (years), mean (SD)		64.6 (11.7)
Gender, n^a^ (%)		
Female		70 (44.3)
Male		79 (50.0)
Other/Prefer not to say		1 (0.6)
Marital status, n (%)		
Married		124 (83.8)
Divorced		9 (6.1)
Separated		3 (2.0)
Single, never married		12 (8.1)
Cancer type, n (%)		
Breast		41 (26.6)
Hematological		32 (20.8)
Gastrointestinal		25 (16.2)
Genitourinary		26 (16.8)
Lung		12 (7.8)
Gynecological		5 (3.2)
NOS^b^		13 (8.4)
Treatment intent, n (%)		
Curative		81 (53.6)
Noncurative		64 (42.4)
Not sure		6 (4.0)
Self-reported functional status, n (%)		
ECOG^c^ ≤1 (good)		128 (83.2)
ECOG ≥2 (poor)		26 (16.8)
Respondent location, n (%)		
Winnipeg chemotherapy sites		83 (53.9)
Winnipeg radiotherapy sites		29 (18.8)
CCPN^d^ sites		42 (27.2)

a Due to missing data for some responses, not all sections sum to 154 responses.

b NOS: not otherwise specified.

c ECOG: Eastern Cooperative Oncology Group functional status scale.

d CCPN: Community Cancer Program Network Site (ie, non-Winnipeg locations).

**Table 2.** Patient-reported outcome measures results, including average scores and number of respondents with above or below average patient-reported experience measures scores (n=154).

PREM^a^_subscale_	Scores, mean (SD)	Respondents, n>mean^b^	Respondents, n<mean^b^
PSCC^c^	80.8 (10.4)	94	60
CCCQP^d^	78.2 (13.4)	79	75
CCCQP^e^_comm_	50.5 (10.1)	80	74
CCCQP^f^_nav_	27.7 (4.7)	77	77

a PREM: patient-reported outcome measures.

b No respondents scores were equal to mean, when calculated to nearest 100th.

c PSCC: Patient Satisfaction with Cancer Care.

d CCCQP: Cancer Care Coordination Questionnaire for Patients.

e CCCQP_comm_: Cancer Care Coordination Questionnaire for Patients, communications subscale.

f CCCQP_nav_: Cancer Care Coordination Questionnaire for Patients, navigation subscale.

**Table 3.** Predictors of coordination of care and satisfaction with care, univariate analysis.

Variable		CCCQP^a^, OR^b^ (95% CI)		CCCQP^c^_comm_, OR (95% CI)		CCCQP^d^_nav_, OR (95% CI)		PSCC^e^, OR (95% CI)	
Female (vs male)^f^		1.01 (0.53‐1.91)		1.07 (0.56‐2.03)		0.85 (0.45‐1.62)		1.19 (0.62‐2.30)	
ECOG^g^ ≥2 (vs ≤1)		0.36^h^ (0.15‐0.88)		0.52 (0.22‐1.23)		0.57 (0.24‐1.35)		0.33^h^ (0.14‐0.78)	
Age (years)									
<60		0.72 (0.34‐1.53)		0.63 (0.30‐1.32)		0.75 (0.36‐1.59)		0.44^h^ (0.20‐0.93)	
60‐69		1.18 (0.59‐2.35)		0.92 (0.47‐1.83)		1.58 (0.79‐3.17)		2.21^h^ (1.05‐4.62)	
>69		1.12 (0.56‐2.26)		1.65 (0.81‐3.35)		0.80 (0.40‐1.61)		0.96 (0.47‐1.96)	
Marital status									
Married		2.09 (0.85‐5.14)		2.09 (0.85‐5.14)		1.96 (0.80‐4.81)		1.49 (0.62‐3.59)	
Divorced		0.24 (0.05‐1.18)		0.24^i^ (0.05‐1.18)		0.25^i^ (0.05‐1.25)		0.46 (0.12‐1.80)	
Separated		1.82 (0.16‐20.47)		1.82 (0.16‐20.47)		0.60 (0.17‐21.63)		1.22 (0.11‐13.80)	
Single, never married		0.62 (0.19‐2.04)		0.62 (0.19‐2.04)		0.65 (0.20‐2.16)		0.84 (0.25‐2.79)	
Location									
Chemotherapy (Winnipeg)		0.72 (0.38‐1.37)		0.84 (0.45‐1.59)		0.66 (0.35‐1.24)		0.70 (0.36‐1.34)	
Radiotherapy (Winnipeg)		1.20 (0.54‐2.63)		1.27 (0.61‐2.64)		1.41 (0.68‐2.93)		0.86 (0.38‐1.90)	
CCPN^j^^k^		1.32 (0.63‐2.74)		0.98 (0.45‐2.17)		1.28 (0.58‐2.81)		1.89 (0.86‐4.16)	
Treatment intent									
Curative		1.26 (0.66‐2.39)		1.47 (0.78‐2.80)		1.27 (0.67‐2.41)		1.20 (0.62‐2.32)	
Noncurative		0.80 (0.42‐1.53)		0.68 (0.36‐1.31)		0.79 (0.41‐1.50)		0.89 (0.46‐1.73)	
Unsure		0.93 (0.18‐4.78)		0.91 (0.18‐4.65)		0.99 (0.19‐5.05)		0.63 (0.12‐3.23)	
Cancer type									
Breast		1.00 (0.49‐2.04)		0.96 (0.47‐1.97)		0.94 (0.46‐1.91)		0.58 (0.28‐1.19)	
Hematological		1.29 (0.59‐2.81)		1.06 (0.49‐2.31)		1.61 (0.73‐3.55)		2.23^i^ (0.93‐5.36)	
Gastrointestinal		1.25 (0.53‐2.97)		1.48 (0.62‐3.53)		1.10 (0.47‐2.59)		2.28^i^ (0.85‐6.09)	
Genitourinary		0.53 (0.23‐1.27)		0.76 (0.33‐1.76)		0.57 (0.24‐1.35)		0.85 (0.36‐1.99)	
Lung		1.36 (0.41‐4.49)		0.92 (0.28‐2.99)		1.44 (0.44‐4.75)		0.89 (0.27‐2.93)	
Gynecological		1.44 (0.23‐8.87)		3.84 (0.42‐35.19)		1.52 (0.25‐9.36)		0.41 (0.07‐2.55)	
NOS^l^		0.80 (0.26‐2.50)		0.55 (0.17‐1.76)		0.60 (0.19‐1.92)		0.52 (0.17‐1.62)	

a CCCQP: Cancer Care Coordination Questionnaire for Patients.

b OR: odds ratio.

c CCCQP_comm_: Cancer Care Coordination Questionnaire for Patients, communications subscale.

d CCCQP_nav_: Cancer Care Coordination Questionnaire for Patients, navigation subscale.

e PSCC: Patient Satisfaction with Cancer Care.

f Gender: responses of “other/prefer not to say” was not included in the analysis due to small numbers (n=1).

g ECOG: Eastern Cooperative Oncology Group functional status scale.

h *P*≤.05.

i *P*≤.10 and >.05.

j CCPN: Community Cancer Program Network Site (ie, rural/non-Winnipeg).

k Except for gender and ECOG, odds ratios for each category level are in comparison to the sum of all other levels in the category (eg, age <60 years vs all other age categories).

l NOS: not otherwise specified.

**Table 4.** Results of stepwise model building (*P*_in_<.10, *P*_out_>.10) for patient satisfaction with care.^a^

Variable	B	OR^b^ (95% CI)		*P* value		
ECOG^c^ ≥2	−1.011	0.364 (0.133‐0.996)		.05		
Age <60 years	−0.844	0.43 (0.194‐0.956)		.04		
Hematologic cancer	1.071	2.917 (1.039‐8.191)		.04		

a *R*2=0.129.

b OR: odds ratio. ORs for each category level are in comparison to all other levels in the category as listed in Table 3 (eg, age <60 years vs all other ages categories).

c ECOG: Eastern Cooperative Oncology Group functional status scale.

### Respondent Characteristics and Response Rate

A total of 203 individual surveys (representing a response rate of 3.3%‐2.0%) were collected between July 31, 2020, and February 28, 2022. Two of these were paper responses, the rest were collected online. One hundred and fifty-four responses were complete for all PREMs and were included in the analysis. The average age of respondents was 65 (SD 11.7) years. The majority were male (n=79, 52.7%), married (n=124, 83.8%), and receiving care from a site in the city of Winnipeg (n=112, 72.7%). Most respondents generally had good performance status (ECOG ≤1, n=128, 83.2%) and were being treated with curative intent (n=81, 53.6%). Breast cancer was the most common cancer type (n=41, 26.6%). Seventy-seven (50%) respondents opted in to be contacted about the associated classic grounded theory study, and 111 (72.1%) opted in to be contacted about research opportunities in general. The average time spent completing the survey was 12 minutes and 49 seconds.

### Patient Demographics, Patient Satisfaction, and Care Coordination

Only age was found to predict patient satisfaction. Univariate analysis demonstrated that age between 60 years and 69 years was a predictor of above-average satisfaction (OR 2.205, 95% CI 1.045‐4.624; *P*=.04). Age below 60 years was identified as a predictor of below-average satisfaction (OR 0.437, 95% CI 0.204‐0.934; *P*=.03). In multivariate analysis, age <60 years remained statistically significant as a predictor, being associated with below-average patient satisfaction (OR –0.844, 95% CI 0.194‐0.956; *P*=.03). Neither marital status nor gender were identified as predictors of the level of satisfaction.

No demographic characteristic was identified to be a statistically significant predictor of below or above average care coordination, including for either of the communication or navigation subscales.

### Functional Status, Disease Characteristics, Patient Satisfaction, and Care Coordination

Poor performance status (ie, ECOG ≥2) was identified as a predictor of below average patient satisfaction both in univariate (OR 0.327, 95% CI 0.137‐0.782, *P*=.01) and in multivariate (OR 0.364, 95% CI 0.133‐0.996, *P*=.049) analysis. In multivariate analysis, hematological malignancies were associated with above-average satisfaction with care (OR 2.917, 95% CI 1.039‐8.191, *P*=.04).

Only functional status was found to be a predictor of care coordination, with decreased functional status predicting below-average experience with coordination of care. Univariate analysis identified ECOG ≥2 as a predictor of below-average care coordination (OR 0.357, 95% CI 0.145‐0.880, *P*=.03). No single disease type was statistically significant for an association with either above or below-average care coordination.

### Treatment Location, Patient Satisfaction, and Care Coordination

Treatment location was not identified to be a statistically significant predictor of either above or below average care coordination or satisfaction.

### Applying Fit Theory

While fit theory is explained in detail elsewhere [1], to assist the reader, a brief explanation of the relevant concepts used for the interpretation of the survey data is presented here. The core concept of fit theory is that the quality of the patient experience is a reflection of the fit between the characteristics of the patient and the available assets of the health care system [1]. Patient characteristics include those that are physical and nonphysical. Examples of physical characteristics include patient location and the characteristics of the cancer being treated [1]. Examples of nonphysical characteristics include a patient’s fears, their spiritual faith system, and the responsibilities they have outside of the patient role (ie, in the nonpatient domain of their life) [1]. Health care assets are divided into biomedical assets and human assets [1]. Biomedical assets are the technical resources available for the diagnosis and treatment of a patient’s cancer. Examples include the technical skills of health care providers and available medications [1]. Human assets represent the tools and resources available for care delivery beyond primarily biomedically directed care, including those required for delivering care that is respectful, promotes dignity, and engenders trust. Examples of human assets include the bedside manner of health care providers and their availability to spend time answering questions [1]. Importantly, informal caregivers (ie, the friends and family of patients who provide unpaid support) can improve the cancer experience by providing additional assets that could improve the fit between the patient’s characteristics and the health care system. Some examples of the consequences of a good fit between patient characteristics and medical assets include the receipt of healthcare requiring minimal disruption to the patient’s life, increased optimism, and decreased impact on informal caregivers [1]. Some examples of consequences of poor fit include anger, anxiety, fear, increased disruption of the aspects of the patient’s life not related to receiving cancer care, and increased reliance on informal caregivers [1].

Age and functional status were both identified through data analysis to be predictive of PREMs outcome with a high degree of confidence and able to be directly connected to concepts defined by fit theory [1]. The relationship between functional status and both satisfaction and care coordination is best explained by the fit between the physical characteristics of the patient and medical assets [1]. Throughout the cancer journey, an individual’s ability to function both mentally and physically can be impacted by treatment-related toxicity and progressive disease. In addition, concomitant and pre-existing comorbidities may impact an individual’s function, making it harder for them to participate in cancer care. Cancer care is complex, often requiring multiple visits for diagnostic and therapeutic interventions, as well as to meet with different specialist teams [1]. These visits can occur at different times, dates, and geographic locations, and patients are ultimately responsible for physically navigating their care. Accessing biomedical assets, including for diagnosis and treatment, often requires travel, waiting in waiting areas for unknown amounts of time, and receiving and following what may be complex and unfamiliar instructions. These activities all require physical and mental ability, which may result in an especially overwhelming and frustrating experience for those already operating at a decreased level of function.

The relationship between age and satisfaction is most directly explained by the nonphysical characteristics of the patient and biomedical assets. In particular, fit theory [1] identifies that roles and responsibilities occurring in the nonpatient domain (eg, involvement in employment, raising children, pursuing education, and caring for older parents) may reduce an individual’s flexibility to participate in care. Patients less than 60 are arguably more likely to be involved in raising children, employment, and multiple other generative social roles, making it more challenging to participate in the patient role with its competing priorities. It is not surprising that the age group between 60‐69 was associated with increased satisfaction with care, as this group is probably more likely to have fewer of the responsibilities related to earlier adult life (eg, more likely to be retired and less likely to have dependent children) and therefore more flexible to participate in medical care. These explanations for why age predicts satisfaction with care and functional status predicts the experience with care coordination are further supported by the fact that in the multivariate model, both age <60 years and poor functional status (ie, ECOG >2) were selected as variables predictive of below-average satisfaction. This suggests that the correlation between age and satisfaction is not purely related to function, but that there is something else specific to younger age that results in poor fit with medical assets.

The survey finding that hematologic cancer is associated with above-average satisfaction with care compared to those with solid tumors is not directly explained by fit theory. Fit theory supports that there is something unique about the cancer experience for this group and that important differences likely exist between the physical and nonphysical characteristics of the hematological and nonhematological cancer patient and the human and biomedical assets available to them through the healthcare system [1]. However, between the data collected here and the insights provided by fit theory [1], a further explanation is not possible.

## Discussion

### Principal Findings

This survey study identified age, functional status, and disease type as predictors of the quality of the patient experience. Specifically, being between the ages of 60 years and 69 years, having good functional status (ie, ECOG ≤1), and being treated for a hematologic malignancy was associated with better satisfaction with care. In contrast, poorer functional status was associated with both decreased satisfaction with care and care coordination. These results are important as they identify specific populations where further exploration of the interaction between patient characteristics and health services delivery is likely to yield opportunities for service improvement.

### Comparison With Previous Work

Compared with other studies exploring patient satisfaction and care coordination, which used the same validated PREMs, the median scores were similar. Studies in the lung [25] and colorectal [21] populations reported CCCQP scores of 78.1 (SD 10.6) and 76.1 (SD 10.9), respectively. In another study that was not specific to a certain tumor group, median PSCC scores of 78.2 (SD 1.92) were reported. The fact that median PREM scores reported in this survey were higher than what has been reported elsewhere (ie, median CCCQP 78.2, SD 13.4, and median PSCC 80.8, SD 10.4) is perhaps reassuring that the impact of COVID-19 on the patients’ experience with care coordination and satisfaction was not significant. However, without a pre-existing baseline from the Manitoba population, this finding is difficult to interpret.

Importantly, the findings of other studies that have used the same, or similar, PREMs [20-23,25,26] in the cancer population support that fit theory is capable of informing meaningful and consistent data interpretation. Specifically, the conclusion that below-average patient satisfaction and care coordination reflect a relative deficiency of fit between health care system assets and the characteristics of the individual patient [1] is an explanation that can be plausibly applied throughout the literature in the cancer context. For instance, in terms of satisfaction, other authors have identified that White race is associated with higher patient satisfaction compared with Hispanic or African American populations [23], suggesting underlying issues with inequity and inequality in terms of available healthcare assets and their fit. Satisfaction has also been identified to be inversely associated with higher levels of fatigue [26], reflecting a relationship between the patient experience, physical function, and symptoms. Similarly, improved experience with care coordination has been found to be associated with higher levels of education [22], more experience with the health system [21], improved health literacy [21], fewer comorbid conditions [21,22], presence of a general practitioner [21], nonrural treatment [21], and being between the ages of 60‐69 years [20]. These variables can all be argued to either impact or reflect the fit between the characteristics of the patient and health care system assets. The ability of fit theory to facilitate meaningful interpretation of data, both from data collected in Manitoba, Canada, as well as from other locations, highlights the value of middle-range theory as a powerful tool for evolving the understanding of the cancer experience.

### Clinical Implications

The findings from this survey support that there is value in collecting cancer-specific PREMs as part of routine cancer care [27], as several patient characteristics were identified that are statistically associated with a poorer-than-average experience with cancer care in Manitoba, Canada. Clinical teams need to be equipped with such data to better identify when individual patients and their informal caregivers are not having a good experience with care. The approach to capturing PREMs by embedding links to a survey in patient materials (ie, their printed personalized treatment schedule) is a simple way to collect such data that does not impose an additional workload on care teams.

The findings of this survey support that those outside of the 60‐69 years age range [28], as well as those with poorer functional status [29], likely face additional challenges with receiving care. Care teams are encouraged to build in specific steps in their clinical processes to assess the care needs of these groups. For instance, taking time to explore what additional supports could be provided to those identified as having poor functional status [1,29] and making efforts to schedule appointments with consideration of both the patient’s and their support team’s other responsibilities in mind (eg, employment, availability of people to provide rides, or transportation services) [1] are simple steps that are likely to enhance the experience of receiving care for the groups identified in this study.

### Research Implications and Future Directions

Unlike some survey recruitment methods, which have response rates of greater than 50% [30,31], the response rate for this survey was very low (ie, estimated to be between 3.3% and 2.0%). This was likely related to the passive approach for recruitment used, as the survey link was simply present on patients’ personalized appointment schedules with relatively little additional information. This is in contrast to survey methods that, although they have higher response rates, require active efforts by researchers and clinicians, including through repeated in-person contact or the coordination of repeated mail-outs [32]. In addition, barriers to survey participation [33], such as limited comfort with completing online surveys or lack of internet access through a computer or smart device, are likely additional factors that contributed to the poor response rate.

Despite the low response rate, the method of incorporating a simple survey link in clinical materials, as was used in this study, demonstrates promise as a recruitment approach for PREM collection, the collection of other survey data, or recruitment for other patient engagement activities. This approach is likely to be useful whenever pragmatic data collection is required, such as in capturing real-world PREM data for quality improvement. Understanding how to increase survey responses using the method applied in this study is an important future direction, as this approach to collecting survey responses requires low resources in terms of researcher and clinician time and has the potential to yield insightful results.

### Limitations

First, the most significant limitation of this survey was the small sample size. While several statistically significant relationships were identified, an increased sample size [24] would have likely resulted in the identification of additional clinically and statistically significant findings. Second, given the low response rate and the fact that nearly 25% of respondents did not complete all survey questions, concerns for bias exist, including selection bias and nonresponse bias [34,35]. As a result of these limitations, the findings presented should be viewed as exploratory and hypothesis-generating.

### Conclusions

This survey study involved collecting PREMs data on the experience of coordination [10] and satisfaction with cancer care [11] using an online survey and a recruitment process that did not interfere with clinical processes. The results of the survey demonstrated important correlations between patient-reported experience with care and the independent variables of age, type of disease, and functional status. The survey results, when examined through the lens of fit theory [1], suggest that there are important variations in how well health care resources can support those living with cancer. In particular, the findings of this survey suggest that further work is needed to better understand how to support younger patients [28] and those with poorer functional status [29,36]. In terms of methods, the approach used in this study for collecting PREMs was relatively simple and can be deployed without increasing the workload of front-line clinical teams. However, further work is needed to improve survey response rates collected in this manner and understand the selection biases associated with this approach.

## Supplementary material

10.2196/58999 Multimedia Appendix 1 Survey tool.

10.2196/58999 Multimedia Appendix 2 Recruitment tools and materials.

10.2196/58999 Checklist 1 Checklist for Reporting Results of Internet E-Surveys.

## Abbreviations

CCCQP: Cancer Care Coordination Questionnaire for Patients
CCCQPcomm: Cancer Care Coordination Questionnaire for Patients, communications subscale
CCCQPnav: Cancer Care Coordination Questionnaire for Patients, navigation subscale
CHERRIES: Checklist for Reporting Results of Internet E-Surveys
ECOG: European Cooperative Oncology Group functional status
HIPAA: Health Information Portability and Accountability Act
OR: odds ratio
PREM: patient-reported experience measure
PSCC: Patient Satisfaction with Cancer Care
