# Supplementary material for: Care Coordination and Patient Satisfaction With Ambulatory Cancer Care During the COVID-19 Pandemic in Manitoba, Canada: Report of An Online Survey Study of Patient-Reported Experience Measures With Interpretation Guided by Fit Theory
Source: JMIR Cancer. 2025 Aug 25;11:e58999. doi: 10.2196/58999 (PMC12377789; doi:10.2196/58999)
Supplement: Multimedia Appendix 1 [file cancer-v11-e58999-s001.pdf]

Impact of COVID-19 on the Patient Experience at CCMB

Introduction

This survey is assessing the changes related to COVID-19 on the experience of patients receiving care through CancerCare Manitoba (CCMB).

Many of the questions are designed for cancer patients. If you are being treated for a non-cancer condition at CCMB please complete the survey anyway. Your responses will be helpful to the researchers.

Only the members of the research team will have access to your responses. Survey results will be presented in a way that you will not be able to be identified as a participant.

This survey is confidential, the answers are being obtained using a system that is compliant with the highest standards for health information security. The risk of a breach of your confidentiality is extremely low, but it is a risk you need to be aware of.

In order to complete this survey you will need to provide you CR#. Your nurse can help you identify this number. Researchers will use this number to gather information from your CancerCare records to help understand the context of your responses.

This survey is for patients 18 years of age and older. If you are not a patient 18 years of age or older and you are interested in participating in this survey please contact the research team using the contact information below.

This study is approved by the University of Manitoba Health Research Ethics Board. (ID: HS23979 - H2020:264)

If you have questions, contact:

Dr. M Thiessen
phone: 204 787 4249
email: mthiessen3@cancercare.mb.ca

Are you 18 years of age or older?

- ○ Yes
- ○ No

You will need to provide your CR# to complete this survey. Your nurse, physician, or the clerk where you checked in can help you locate this number.

Please enter your CR#:

____

Impact of COVID-19 on the Patient Experience at CCMB

Patient Satisfaction with Care

For the following questions, please consider your experience with receiving care since the implementation of changes related to COVID-19 (i.e. March 2020).

I felt that my health concerns were understood.

- ◯ Strongly Agree
- ◯ Agree
- ◯ Neutral
- ◯ Disagree
- ◯ Strongly Disagree

I felt that I was treated with courtesy and respect.

- ◯ Strongly Agree
- ◯ Agree
- ◯ Neutral
- ◯ Disagree
- ◯ Strongly Disagree

I felt included in decisions about my health.

- ◯ Strongly Agree
- ◯ Agree
- ◯ Neutral
- ◯ Disagree
- ◯ Strongly Disagree

I was told how to take care of myself.

- ◯ Strongly Agree
- ◯ Agree
- ◯ Neutral
- ◯ Disagree
- ◯ Strongly Disagree

I felt encouraged to talk about my personal health concerns.

- ◯ Strongly Agree
- ◯ Agree
- ◯ Neutral
- ◯ Disagree
- ◯ Strongly Disagree

I felt I had enough time with my doctor.

- ◯ Strongly Agree
- ◯ Agree
- ◯ Neutral
- ◯ Disagree
- ◯ Strongly Disagree

My questions were answered to my satisfaction.

- ◯ Strongly Agree
- ◯ Agree
- ◯ Neutral
- ◯ Disagree
- ◯ Strongly Disagree

Making an appointment was easy.

- ◯ Strongly Agree
- ◯ Agree
- ◯ Neutral
- ◯ Disagree
- ◯ Strongly Disagree

I knew what the next step in my care would be.

- ◯ Strongly Agree
- ◯ Agree
- ◯ Neutral
- ◯ Disagree
- ◯ Strongly Disagree

I feel confident in how I deal with the health care system.

- Strongly Agree
- Agree
- Neutral
- Disagree
- Strongly Disagree

I was able to get the advice I needed about my health issues.

- Strongly Agree
- Agree
- Neutral
- Disagree
- Strongly Disagree

I knew who to contact when I had a question.

- Strongly Agree
- Agree
- Neutral
- Disagree
- Strongly Disagree

I received all the services I needed.

- Strongly Agree
- Agree
- Neutral
- Disagree
- Strongly Disagree

I am satisfied with the care I received.

- Strongly Agree
- Agree
- Neutral
- Disagree
- Strongly Disagree

The doctors seemed to communicate well about my care.

- ◯ Strongly Agree
- ◯ Agree
- ◯ Neutral
- ◯ Disagree
- ◯ Strongly Disagree

I received high-quality care from my regular doctor.

- ◯ Strongly Agree
- ◯ Agree
- ◯ Neutral
- ◯ Disagree
- ◯ Strongly Disagree

I received high-quality care from my specialists.

- ◯ Strongly Agree
- ◯ Agree
- ◯ Neutral
- ◯ Disagree
- ◯ Strongly Disagree

My regular doctor was informed about the results of the tests I got.

- ◯ Strongly Agree
- ◯ Agree
- ◯ Neutral
- ◯ Disagree
- ◯ Strongly Disagree

Impact of COVID-19 on the Patient Experience at CCMB

Care Coordination

The following questions ask about the care that you have received from <u>all</u> the health professionals who have been looking after you during your treatment for cancer.
The health professionals include anyone who has been involved in your care such as a surgeon, GP, medical oncologist, radiation oncologist, respiratory physicain, care coordinator, cancer nurse, community nurses, palliative care, social worker, stomal therapist, physiotherapist, psychologist or counsellor.

When answering the below questions we would like you to think about your experience with the health system and how well you feel your care was organised and coordinated between all the different health professionals you have seen as part of your care since implementation of changes related to COVID-19 (i.e. March 2020).

I knew the warning signs and symptoms I should watch for to monitor my health.

- ◯ Strongly Disagree
- ◯ Disagree
- ◯ Neutral
- ◯ Agree
- ◯ Strongly Agree

I always knew what tests, treatments and follow up were planned for me.

- ◯ Strongly Disagree
- ◯ Disagree
- ◯ Neutral
- ◯ Agree
- ◯ Strongly Agree

I always knew the reason why I was having a test or treatment.

- ◯ Strongly Disagree
- ◯ Disagree
- ◯ Neutral
- ◯ Agree
- ◯ Strongly Agree

I knew which therapies were suitable for me (eg. surgery, chemotherapy, radiotherapy).

- Strongly Disagree
- Disagree
- Neutral
- Agree
- Strongly Agree

I was fully informed about the benefits and harms of any treatments.

- Strongly Disagree
- Disagree
- Neutral
- Agree
- Strongly Agree

I had access to all the additional services that I needed (eg. physiotherapy, counselling, cancer support groups, social worker support, pain management, palliative care, nutritional advice, stoma therapy).

- Strongly Disagree
- Disagree
- Neutral
- Agree
- Strongly Agree

I had a good understanding of what I was responsible for to help my treatment plan run smoothly.

- Strongly Disagree
- Disagree
- Neutral
- Agree
- Strongly Agree

I had sufficient help from health professionals with dealing with the emotional impact of my cancer.

- Strongly Disagree
- Disagree
- Neutral
- Agree
- Strongly Agree

I had sufficient help from health professionals with practical arrangements such as organizing transport, accommodation and appointments.

- Strongly Disagree
- Disagree
- Neutral
- Agree
- Strongly Agree

I was fully informed by health professionals about my financial entitlements (eg. Medicare and health fund claims, travel allowances).

- Strongly Disagree
- Disagree
- Neutral
- Agree
- Strongly Agree

The health professionals looking after me always picked up on whether I was feeling anxious or down.

- Strongly Disagree
- Disagree
- Neutral
- Agree
- Strongly Agree

Coordination of Care

The following questions ask about your experience with different members of your healthcare team.

My doctors always asked how well my family and I were coping.

- ◯ Strongly Disagree
- ◯ Disagree
- ◯ Neutral
- ◯ Agree
- ◯ Strongly Agree

My doctors always asked how my visits with other health professionals were going.

- ◯ Strongly Disagree
- ◯ Disagree
- ◯ Neutral
- ◯ Agree
- ◯ Strongly Agree

I always knew who to contact if I had concerns about my health or treatment plan.

- ◯ Strongly Disagree
- ◯ Disagree
- ◯ Neutral
- ◯ Agree
- ◯ Strongly Agree

I always knew who to call out of business hours if I had a problem.

- ◯ Strongly Disagree
- ◯ Disagree
- ◯ Neutral
- ◯ Agree
- ◯ Strongly Agree

I was confused about the roles of the different health professionals involved in my care.

- ◯ Strongly Disagree
- ◯ Disagree
- ◯ Neutral
- ◯ Agree
- ◯ Strongly Agree

It was difficult to meet the financial costs associated with my health care.

- ◯ Strongly Disagree
- ◯ Disagree
- ◯ Neutral
- ◯ Agree
- ◯ Strongly Agree

The health professionals looking after me were not always fully informed about my history and progress.

- ◯ Strongly Disagree
- ◯ Disagree
- ◯ Neutral
- ◯ Agree
- ◯ Strongly Agree

I never had any difficulty getting an appointment with my GP.

- ◯ Strongly Disagree
- ◯ Disagree
- ◯ Neutral
- ◯ Agree
- ◯ Strongly Agree

I never had to wait too long to get the first available appointment for a test or treatment.

- ◯ Strongly Disagree
- ◯ Disagree
- ◯ Neutral
- ◯ Agree
- ◯ Strongly Agree

Impact of COVID-19 on the Patient Experience at CCMB

Questions about the different ways physicians have interacted with patients.

Since the changes in healthcare delivery related to COVID-19 began in Manitoba (i.e. March 2020):

How many in-person doctors visits have you had? (i.e. not over the phone or over video-conferencing)

- ◯ 0
- ◯ 1 - 4
- ◯ 5 - 8
- ◯ 9 or more

How would you rate your experience with in-person clinic visits with physicians through CancerCare.

1 (Very Poor)	2	3	4	5	6	7	8	9	10 (Excellent)	N/A
★	★	★	★	★	★	★	★	★	★	◯

How many doctors visits over the phone have you had?

- ◯ 0
- ◯ 1 - 4
- ◯ 5 - 8
- ◯ 9 or more

How would you rate your phone visits with CancerCare physicians?

1 (Very Poor)	2	3	4	5	6	7	8	9	10 (Excellent)	N/A
★	★	★	★	★	★	★	★	★	★	◯

How many doctors visits through Telehealth (i.e. through a video conference where you had to visit a clinic to be connected with a physician in another location) have you had?

- ◯ 0
- ◯ 1 - 4
- ◯ 5 - 8
- ◯ 9 or more

How would you rate your experience with Telehealth clinic visits with CancerCare physicians

1 (Very Poor)	2	3	4	5	6	7	8	9	10 (Excellent)	N/A
★	★	★	★	★	★	★	★	★	★	○

How many doctors visits using non-Telehealth video-conferencing have you had? (i.e. through a video conference where you were able to connect with a physician through a personal smartphone or laptop using software such as Zoom, Microsoft Teams, Skype, or Blue Jeans).

- ○ 0
- ○ 1 - 4
- ○ 5 - 8
- ○ 9 or more

How would you rate your experience with non-Telehealth video-conferencing?

1 (Very Poor)	2	3	4	5	6	7	8	9	10 (Excellent)	N/A
★	★	★	★	★	★	★	★	★	★	○

Impact of COVID-19 on the Patient Experience at CCMB

Demographic Questions

What is your postal code?

When were you born?

Please enter your birth date:

Date

MM/DD/YYYY

What is your gender?

- ◯ Male
- ◯ Female
- ◯ Prefer not to answer
- ◯ Other (please specify)

What is your current marital status?

- ◯ Married and/or living with a partner
- ◯ Divorced
- ◯ Separated
- ◯ Single, never married

What best describes the intent of your care at CancerCare:

- ◯ part of a strategy to cure the condition
- ◯ part of a strategy to control (not cure) the condition
- ◯ not sure

What is the name of the condition that is being managed by your healthcare team at CancerCare Manitoba? (e.g. lung cancer, breast cancer, hemophilia)

This condition was first diagnosed:

- ◯ After March 2020
- ◯ Between March 2020 and six months ago
- ◯ between six and twelve months ago
- ◯ between 1 and 2 years ago
- ◯ more than 2 years ago

I would describe myself as:

- ◯ Fully active, able to carry on all pre-disease performance without restriction
- ◯ Restricted in physically strenuous activity, but ambulatory and able to carry out work of a light and sedentary nature (e.g. light house work, office work)
- ◯ Ambulatory and capable of all self-care but unable to carry out any work activities. Up and about more than 50% of waking hours.
- ◯ Capable of only limited self-care, confined to bed or chair more than 50% of waking hours
- ◯ Completely disabled. Cannot carry on any self-care. Totally confined to bed or chair

Would you be willing to be contacted in the future about participating in research opportunities related to the patient experience, in general?

- ◯ Yes
- ◯ No

Impact of COVID-19 on the Patient Experience at CCMB

Would you be willing to be contacted by researchers about participating in studies to help improve the experience of patients who receive physician care through telemedicine (i.e. Telehealth, phone contact, and/or video-conferencing)?

- ◯ Yes
- ◯ No

Impact of COVID-19 on the Patient Experience at CCMB

Consent to by contacted for research opportunities.

By providing the following information you consent to having your responses to this survey reviewed by the researchers conducting this survey in order to connect you with other researchers working with CCMB. Your responses will not be shared, but you could be contacted about research opportunities that you may be eligible for.

All research opportunities are voluntary. You are not obligated to participate in any opportunities you are contacted about.

Please provide your name, phone number, and/or email address so that you can be contacted by researchers in the future.

First and Last Name __________

Email Address __________

Phone Number __________

How would you prefer to be contacted about participating in research?

- ○ Email
- ○ Phone
