# Supplementary material for: Care Coordination and Patient Satisfaction With Ambulatory Cancer Care During the COVID-19 Pandemic in Manitoba, Canada: Report of An Online Survey Study of Patient-Reported Experience Measures With Interpretation Guided by Fit Theory
Source: JMIR Cancer. 2025 Aug 25;11:e58999. doi: 10.2196/58999 (PMC12377789; doi:10.2196/58999)
Supplement: Multimedia Appendix 2 [file cancer-v11-e58999-s002.pdf]

Appendix 2: Recruitment Materials

A) Invitation Cards:

Front

15 minute online survey
How have COVID-19-related changes at CancerCare Manitoba impacted **you?**

Go to:
_________.healthsurveys.ca
Username: **Manitoba**
Password: **Summer2020**

CancerCare Manitoba
ActionCancerManitoba

Back

For more information:
Dr. M. Thiessen
mthiessen3@cancercare.mb.ca
204-787-4249

B) Patient Schedule:

Visit Schedule
Sat Nov 14, 2020

Patient Testpatient, Deborah-Lee
Date of Birth Sep 2, 1992
CR # T25091992

Printout from Manager

Address Deborah-Lee Testpatient
123 Honeysuckle Bay
Winnipeg, Manitoba R3M1B1

Institution CancerCare MB - St. Boniface Unit
Telephone (204)237-2033
Address 409 Tache Avenue
Winnipeg Manitoba Canada R2H2A6

Date Sat Nov 14, 2020
Visit Provider James TESTphysician
Time / Event 08:00 AM IV Systemic Medicati Chair 07 J TESTphysician
Chair 07 location: Treatment Room Physical distancing may not permit a companion to attend all visits, if required please call your clinic.
How has your care been during COVID19? Complete our online survey at: http://stb.healthsurveys.ca

C) Poster:

Have 15 Minutes?

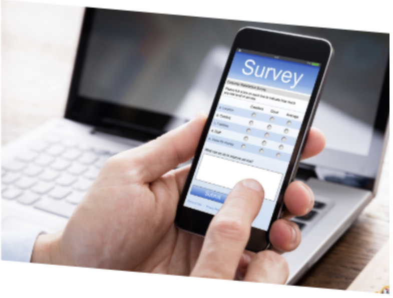

We want to know how changes at CancerCare Manitoba related to COVID-19 have impacted your experience.

Help us by completing a 15-minute online survey.

To complete the survey:

1. Use your smart device/computer to go to: **"_________.healthsurveys.ca"**
2. Enter:
   Username: Manitoba
   Password: Summer2020
3. Click on the link!

Protip:
Take a picture of this poster and use it to access the survey while waiting for appointments or treatment.

If you have any questions or concerns, please contact:

Dr. M. Thiessen
Phone: 204-787-4249
Email: mthiessen3@cancercare.mb.ca

Approved by CancerCare Manitoba's Research Resource Impact Committee

CancerCare Manitoba
ActionCancerManitoba
